# Editorial: PTCY and Allo-HCT: a deep dive into outcomes, toxicities, and patient-centered care

**DOI:** 10.3389/fimmu.2026.1991757

**Published:** 2026-09-29

**Authors:** Fotios V. Michelis, Carmen Martinez, Maria Queralt Salas

**Affiliations:** 1 Hans Messner Allogeneic Transplant Program, Princess Margaret Cancer Centre, University Health Network, Toronto, ON, Canada; 2 Hematopoietic Transplantation Unit, Hematology Department, ICAMS, Hospital Clínic de Barcelona, Barcelona, Spain

**Keywords:** allogeneic hematopoietic cell transplantation, graft-versus- host disease, outcomes, post-transplant cyclophosphamide (PTCy), toxicity

## Introduction

Post-transplant cyclophosphamide (PTCy) has reshaped the landscape of allogeneic hematopoietic cell transplantation (allo-HCT) over the past two decades. What began as a strategy to make haploidentical transplantation feasible has become a near-universal platform for graft-versus-host disease (GVHD) prophylaxis, now extending into matched related, matched unrelated, and mismatched unrelated donor settings. By selectively eliminating alloreactive proliferating T cells while sparing regulatory populations, PTCy has narrowed, and in many centers erased, the outcome gap that once separated haploidentical from fully matched transplantation, dramatically expanding donor access for patients without a matched sibling or unrelated donor. Yet this success has introduced its own follow-up questions: how conditioning intensity, donor characteristics, and immunosuppressive partners interact with PTCy; how the alkylating burden of cyclophosphamide itself drives organ toxicity; and how clinicians can better protect immune reconstitution, infection risk, and quality of life once GVHD is controlled. The eight studies included in our published Research Topic “PTCy and Allo-HCT: A Deep Dive into Outcomes, Toxicities, and Patient-Centered Care” collectively investigate these questions, moving the field from “does PTCy work” to “how do we optimize it.”

## Clinical outcomes, graft sources, and donor selection in the PTCy era

Three contributions refine how conditioning and donor variables shape outcomes when PTCy is used as the prophylactic backbone. Pizzano et al. compared calcineurin inhibitor/mycophenolate mofetil with sirolimus/mycophenolate mofetil platforms in PTCy haploidentical transplantation and applied the transplant conditioning intensity (TCI) score, showing that survival was essentially superimposable between immunosuppressive combinations, while higher conditioning intensity independently predicted severe acute and chronic GVHD—reframing conditioning intensity, not just the GVHD-prophylaxis drug, as a modifiable toxicity lever. Pinedo et al. extended PTCy-based allo-HCT into older adults, demonstrating comparable engraftment, toxicity, and non-relapse mortality across age strata up to 65 years and beyond, even as relapse incidence rose with age—evidence that chronological age alone should no longer be a barrier to transplant referral. Complementing this, the multicenter GETH-TC analysis by Munárriz et al. identified donor age of 30 years or older as an independent predictor of inferior overall survival and higher non-relapse mortality in haploidentical transplantation for acute myeloid leukemia, arguing that when multiple haploidentical relatives are available, younger donor age should be prioritized. Together, these studies suggest that the PTCy platform has matured enough that fine-grained donor and conditioning selection, rather than platform choice alone, may drive incremental outcome gains.

## Toxicity mitigation: GVHD, organ injury, and infectious complications

A second group of studies addresses the toxicities that accompany PTCy’s alkylating mechanism and lymphodepletion. Samuels et al. characterized fluid overload arising from hyperhydration used to prevent PTCy-associated hemorrhagic cystitis, finding that nearly all patients developed some degree of fluid overload and that severe grades independently predicted worse non-relapse mortality and survival—positioning early weight gain as a simple, modifiable warning sign. Bailén et al. turned to the liver, showing that transient elastography–measured liver stiffness at day +14 predicted veno-occlusive disease and event-free survival, offering a non-invasive tool to flag hepatic vulnerability in PTCy recipients. Two studies directly compared PTCy with anti-thymocyte globulin (ATG) as competing lymphodepleting strategies in matched unrelated donor transplantation. Aydin et al. found that PTCy was associated with faster CD4+ T-cell recovery and fewer viral infections and cytomegalovirus reactivations than ATG, despite a higher early bacterial infection burden tied to prolonged neutropenia. Furnari et al. reinforced this immunologic narrative, showing that achieving immune reconstitution—more common and earlier with PTCy—independently predicted survival and eliminated infectious mortality, although the advantage of PTCy over ATG was substantially attenuated once clinically significant GVHD itself was accounted for, underscoring that GVHD and its treatment, not the prophylactic agent per se, are the dominant drivers of delayed immune recovery. Together, these papers reveal that toxicity mitigation with PTCy is not a single problem but a constellation of overlapping risks—renal/fluid, hepatic, and immunologic—each amenable to targeted surveillance.

## Toward precision and patient-centered PTCy

A final thread points toward individualizing PTCy itself. Shen et al. demonstrated substantial interpatient variability in cyclophosphamide and mycophenolate mofetil pharmacokinetics, with higher metabolite exposure correlating with slower lymphocyte recovery, suggesting that fixed weight-based dosing may not serve all patients equally and that pharmacokinetic or biomarker-guided dosing could better balance GVHD prevention against immune recovery and, ultimately, infection risk and quality of life.

## Conclusion and future directions

Collectively, this Research Topic illustrates that PTCy has moved from a novel intervention to a mature, adaptable platform whose remaining challenges are ones of refinement: matching conditioning intensity and donor characteristics to the individual patient, anticipating and monitoring fluid, hepatic, and immunologic toxicity before it becomes clinically overt, and eventually personalizing drug exposure itself. A unifying message emerges—early, dynamic biomarkers, whether weight change, liver stiffness, donor age, or drug pharmacokinetics, consistently outperform static, one-size-fits-all risk stratification. Future work should prioritize prospective validation of these biomarkers in randomized or harmonized multicenter cohorts, deeper mechanistic study of how conditioning intensity and immunosuppressive platform interact with alloreactivity, and trials of biomarker- or pharmacokinetically-guided PTCy dosing. Ultimately, extending PTCy’s benefits to older, more diverse, and higher-risk populations while safeguarding organ function, immune competence, and quality of life will define the next decade of allo-HCT research.

